# Taxonomic composition and biodiversity of the gut microbiome from patients with irritable bowel syndrome, ulcerative colitis, and asthma

**DOI:** 10.18699/VJ21.100

**Published:** 2021-12

**Authors:** A.Y. Tikunov, A.N. Shvalov, V.V. Morozov, I.V. Babkin, G.V. Seledtsova, I.O. Voloshina, I.P. Ivanova, A.V. Bardasheva, V.V. Morozova, V.V. Vlasov, N.V. Tikunova

**Affiliations:** Institute of Сhemical Biology аnd Fundamental Medicine of the Siberian Branch of the Russian Academy of Sciences, Novosibirsk, Russia; State Research Center of Virology and Biotechnology “Vector”, Rospotrebnadzor, Koltsovo, Novosibirsk region, Russia; Institute of Сhemical Biology аnd Fundamental Medicine of the Siberian Branch of the Russian Academy of Sciences, Novosibirsk, Russia; Institute of Сhemical Biology аnd Fundamental Medicine of the Siberian Branch of the Russian Academy of Sciences, Novosibirsk, Russia; Institute of Fundamental and Clinical Immunology, Novosibirsk, Russia; Institute of Сhemical Biology аnd Fundamental Medicine of the Siberian Branch of the Russian Academy of Sciences, Novosibirsk, Russia; Institute of Fundamental and Clinical Immunology, Novosibirsk, Russia; Institute of Сhemical Biology аnd Fundamental Medicine of the Siberian Branch of the Russian Academy of Sciences, Novosibirsk, Russia; Institute of Сhemical Biology аnd Fundamental Medicine of the Siberian Branch of the Russian Academy of Sciences, Novosibirsk, Russia; Institute of Сhemical Biology аnd Fundamental Medicine of the Siberian Branch of the Russian Academy of Sciences, Novosibirsk, Russia; Institute of Сhemical Biology аnd Fundamental Medicine of the Siberian Branch of the Russian Academy of Sciences, Novosibirsk, Russia

**Keywords:** microbiome, 16S rRNA sequences, ulcerative colitis, irritable bowel syndrome, bronchial asthma, микробиом, 16S рРНК-последовательности, язвенный колит, синдром раздраженного кишечника, бронхиальная астма

## Abstract

To date, the association of an imbalance of the intestinal microbiota with various human diseases, including both diseases of the gastrointestinal tract and disorders of the immune system, has been shown. However, despite the huge amount of accumulated data, many key questions still remain unanswered. Given limited data on the composition of the gut microbiota in patients with ulcerative colitis (UC) and irritable bowel syndrome (IBS) from different parts of Siberia, as well as the lack of data on the gut microbiota of patients with bronchial asthma (BA), the aim of the study was to assess the biodiversity of the gut microbiota of patients with IBS, UC and BA in comparison with those of healthy volunteers (HV). In this study, a comparative assessment of the biodiversity and taxonomic structure of gut microbiome was conducted based on the sequencing of 16S rRNA genes obtained from fecal samples of patients with IBS, UC, BA and volunteers. Sequences of the Firmicutes and Bacteroidetes types dominated in all samples studied. The third most common in all samples were sequences of the Proteobacteria type, which contains pathogenic and opportunistic bacteria. Sequences of the Actinobacteria type were, on average, the fourth most common. The results showed the presence of dysbiosis in the samples from patients compared to the sample from HVs. The ratio of Firmicutes/Bacteroidetes was lower in the IBS and UC samples than in HV and higher the BA samples. In the samples from patients with intestinal diseases (IBS and UC), an increase in the proportion of sequences of the Bacteroidetes type and a decrease in the proportion of sequences of the Clostridia class, as well as the Ruminococcaceae, but not Erysipelotrichaceae family, were found. The IBS, UC, and BA samples had signif icantly more Proteobacteria sequences, including Methylobacterium, Sphingomonas, Parasutterella, Halomonas, Vibrio, as well as Escherichia spp. and Shigella spp. In the gut microbiota of adults with BA, a decrease in the proportion of Roseburia, Lachnospira, Veillonella sequences was detected, but the share of Faecalibacterium and Lactobacillus sequences was the same as in healthy individuals. A signif icant increase in the proportion of Halomonas and Vibrio sequences in the gut microbiota in patients with BA has been described for the f irst time.

## Abstract (ru)

К настоящему времени показана ассоциация дисбаланса кишечной микробиоты с различными заболе- ваниями человека, включая не только патологии желудочно-кишечного тракта, но и нарушения иммунной системы. Однако, несмотря на значительный объем накопленных данных, многие ключевые вопросы до сих пор остаются без ответа. Описаны различия в микробных сообществах кишечника в зависимости от возраста больных, типа питания и региона проживания. Учитывая ограниченность данных о составе микробиоты кишечника при язвенном колите (ЯК) и синдроме раздраженного кишечника (СРК) у пациентов из регионов Сибири, а также отсутствие сведений о ки- шечной микробиоте больных бронхиальной астмой (БА), цель исследования – оценка биоразнообразия кишечного микробиома пациентов с СРК, ЯК и БА в сравнении с таковым здоровых добровольцев (ЗД). Проведена сравнитель- ная оценка биоразнообразия и таксономической структуры микробиома содержимого кишечника пациентов с СРК, ЯК, БА и ЗД, определенных на основании 16S рРНК-последовательностей бактериальных генов. В четырех выборках доминировали последовательности типов Firmicutes и Bacteroidetes. Третьи по встречаемости во всех группах – по- следовательности типа Proteobacteria, членами которого являются патогенные и условно-патогенные бактерии. По- следовательности типа Actinobacteria были в среднем четвертыми по встречаемости. Результаты показали наличие дисбиоза в образцах пациентов по сравнению со здоровыми участниками. Соотношение Firmicutes/Bacteroidetes в выборках СРК и ЯК уменьшилось относительно ЗД, а в группе БА – увеличилось. В образцах пациентов с заболева- ниями кишечника (СРК и ЯК) обнаружено увеличение доли последовательностей типа Bacteroidetes и уменьшение доли последовательностей класса Clostridia, а также семейства Ruminococcaceae, но не Erysipelotrichaceae. В вы- борках СРК, ЯК и БА отмечено достоверно больше в сравнении с ЗД последовательностей Proteobacteria, включая Methylobacterium, Sphingomonas, Parasutterella, Halomonas, Vibrio, а также последовательности Escherichia и Shigella. В кишечном микробиоме взрослых пациентов с БА выявлено уменьшение доли последовательностей Roseburia, Lachnospira, Veillonella, однако доля последовательностей Faecalibacterium и Lactobacillus была такой же, как и у здо- ровых участников. Впервые получены данные о существенном увеличении доли последовательностей Halomonas и Vibrio в кишечном микробиоме пациентов с бронхиальной астмой.

## Introduction

К настоящему времени накоплено достаточно данных о микробных сообществах кишечника человека и показана ассоциация дисбаланса кишечной микробиоты с раз- личными патологическими состояниями, в том числе не только заболеваниями желудочно-кишечного тракта, но и нарушениями иммунной системы (O’Hara, Shanahan, 2006). Однако, несмотря на значительный объем инфор- мации, многие ключевые вопросы остаются без ответа. Так, до сих пор неизвестно, являются ли такие заболевания кишечника, как язвенный колит (ЯК) и синдром раздраженного кишечника (СРК), результатом нарушенного иммунного ответа на нормальную микробиоту или служат проявлением нормального иммунного ответа на нарушения в микрофлоре кишечника (Cheng, Fisher, 2017).

Патогенез этих заболеваний также не вполне ясен: вероятно, механизм развития имеет сложную природу и опосредован нарушениями кишечной микробиоты, генетической предрасположенностью и экологическими фак- торами (Shen et al., 2018). Известно лишь, что при ЯК и СРК снижено биоразнообразие микробиоты кишечника (Machiels et al., 2014; Dubinsky, Braun, 2015). При ЯК отмечено уменьшение количества представителей типов Bacteroidetes и Firmicutes; для последнего зарегистриро- вано уменьшение встречаемости последовательностей Roseburia hominis и Faecalibacterium prausnitzii (отряд Clostridia, сем. Lachnospiraceae и Ruminococcaceae соот- ветственно) (Machiels et al., 2014; Тикунов и др., 2020). Особенно заметно снижение Akkermansia muciniphila (тип Verrucomicrobia), в норме достигающей 1–5 % всего бактериального сообщества (Manichanh et al., 2012; Bajer et al., 2017). Одновременно в микробиоте пациентов с ЯК увеличено количество представителей Actinomycetes и Proteobacteria, а среди протеобактерий часто выявляют Helicobacter spp., Salmonella spp., Yersinia spp. и энтероин- вазивные Escherichia coli (Saebo et al., 2005; Gradel et al., 2009; Sonnenberg, Genta, 2012; Shen et al., 2018; Тикунов и др., 2020). В случае СРК, как и при ЯК, в микробиоте кишечника количество представителей типа Proteobacteria увеличено, а число представителей Actinomycetes, наобо- рот, уменьшено (Bennet et al., 2015; Su et al., 2018).

Соотношение основных представителей микробного сообщества кишечника человека, Firmicutes/Bacteroidetes, при СРК может и увеличиваться, и уменьшаться (Tana et al., 2010; Rajilić-Stojanović et al., 2011; Jeffery et al., 2012; Jalanka-Tuovinen et al., 2014; Pozuelo et al., 2015; Tap et al., 2017). Такое несовпадение данных может быть связано как с динамичностью кишечного микробного сообщества у отдельного индивидуума, так и высоким биоразнообрази- ем кишечной микробиоты у людей не только при различ- ном состоянии здоровья, но и в зависимости от возраста, региона проживания и особенностей питания (Fujimura et al., 2010; Qin et al., 2010; Donaldson et al., 2016). В связи с этим изучение ассоциации особенностей микробиоты с различными заболеваниями человека – одно из наиболее актуальных направлений современных биомедицинских исследований.

Учитывая ограниченность данных о составе микробиоты кишечника при ЯК и СРК у пациентов из регионов Сибири, а также отсутствие сведений о кишечной микро- биоте больных бронхиальной астмой (БА), цель исследо- вания – оценка 16S рРНК-профилей пациентов с СРК, ЯК и БА в сравнении с таковыми здоровых доноров.

## Материалы и методы

В работе использовали образцы фекалий, полученные от восьми пациентов с БА, восьми больных с СРК и 18 па- циентов с ЯК. Среди исследуемых женщины составили 47.4 %, мужчины – 52.6 %. Диагнозы «синдром раздражен- ного кишечника» и «язвенный колит» подтверждали на основании результатов исследования уровня фекального кальпротектина, данных фиброколоноскопии и гистологического исследования биоптатов, взятых из разных отделов толстой и подвздошной кишок. Образцы пациентов, в которых рутинными методами были обнаружены Clostridium diff icile, не исследовали. Диагноз «атопиче- ская бронхиальная астма» подтверждали на основании общего, биохимического и иммунологического анализов крови и данных сенсибилизации. Также в исследовании использованы восемь образцов здоровых добровольцев без хронических заболеваний и не болевших последние три месяца. Все пациенты и добровольцы отрицали систе- матическое употребление алкоголя, только три больных мужского пола с СРК употребляли табачные изделия. Все участники исследования в течение как минимум двух не- дель до забора образцов не принимали антибактериальные препараты. Все пациенты и добровольцы предоставили информированное согласие с проводимым исследова- нием и анонимной обработкой данных. Исследование одобрено локальным этическим комитетом Автономной некоммерческой организации «Центр новых медицинских технологий в Академгородке».

Выделение суммарной ДНК проводили, как описано ранее (Тикунов и др., 2020). Основные этапы включали осветление 50 мг из каждого образца с последующим при- менением набора для выделения ДНК из клеток тканей и крови (ООО «БиоЛабМикс», Россия) с добавлением лизо- цима для повышения эффективности получения ДНК из грамположительных бактерий. Амплификацию фрагмента гена 16S рРНК, содержащего вариабельные участки V3 и V4, проводили методом полимеразной цепной реакции с использованием в качестве матрицы полученной ДНК фьюжн-праймеров (NEB-FF 5′-ACACTCTTTCCCTACA CGACGCTCTTCCGATCTCTACGGGAGGCAGCAG-3′, NEB-FR 5′-GTGACTGGAGTTCAGACGTGTGCTCTTC CGATCTGGACTACCGGGGTATCT-3′) и высокоточной полимеразы Q5 (New England Biolabs, США). Продукты амплификации очищали электрофоретически в геле из легкоплавкой SeaKem GTG-агарозы (Lonza, США).

Конструирование библиотек выполняли, как описано ранее (Тикунов и др., 2020). Основные этапы включали обогащение полученных ампликонов, введение баркодов и служебных последовательностей с использованием по- лимеразы Q5 и набора олигонуклеотидов Dual index set (New England Biolabs, США) с последующей очисткой полученных библиотек на магнитных частицах AMPure XP (Beckman Coulter, США). Концентрацию ДНК в библиотеках измеряли с помощью набора Qubit dsDNA HS (Life Technologies, США). По результатам измерений библиотеки объединяли в пул таким образом, чтобы соотношение ДНК-библиотек в пуле было эквимолярным Секвенирование вели на платформе MiSeq с использованием набора реагентов MiSeq reagent kit v2 2 × 250-cycles (Illumina, США).

Методы анализа данных секвенирования описаны ранее (Тикунов и др., 2020). Предварительно из последователь- ностей ридов удаляли последовательности адаптеров и проводили фильтрацию ридов по качеству. Полученные риды анализировали с помощью генерации операционных таксономических единиц (OTU) с последующим картированием последовательностей на полученные OTU в пакете программ Usearch-9.2 и использованием класси- фикации ридов алгоритмом Kraken по базе данных из- вестных последовательностей 16S рРНК Silva v.132 (full).

В первом случае OTU генерировали алгоритмом unoise2 с отбраковкой химерных последовательностей и учетом ошибок чтения. Таблицы полученных частот встречае- мости OTU обработаны в среде R3.3.3. Во втором случае полученные риды картировали на базу данных 16S рРНК Silva с помощью алгоритма seed-kraken с использованием разреженного k-мера со специальной решеткой, позволя- ющей увеличить специфичность классификации. Индекс Шеннона рассчитывали в пакете программ R; достовер- ность различий между индексами Шеннона определяли с помощью t-теста Хатчесона. Визуализацию результатов анализа библиотек последовательностей методом главных координат PCoA проводили на основе матриц дистанций с применением пакета программ vegan. Для установления достоверности различий между величинами использовали критерий Стьюдента

## Результаты и обсуждение

Оценка биоразнообразия бактериальных сообществ

На основе ДНК, выделенной из образцов фекалий па- циентов и доноров, сконструированы 42 библиотеки фрагментов гена 16S рРНК. Фрагменты содержали вариабельные участки V3 и V4 гена 16S рРНК – на основе этой последовательности возможна таксономическая класси- фикация большинства бактерий (Chakravorty et al., 2007; Wang, Qian, 2009). Образцы сгруппированы в четыре выборки: БА – 8 образцов больных атопической БА (сред- ний возраст 38.1 года; от 25 до 56 лет), СРК – 8 образцов пациентов с СКР (средний возраст 44.9 года; от 25 до 64 лет), ЯК – 18 образцов пациентов с ЯК (средний возраст 39.6 года; от 25 до 65 лет), здоровые добровольцы (ЗД) – 8 образцов здоровых добровольцев (средний возраст 27.1 года; от 20 до 39 лет). Средний возраст добровольцев в группе ЗД был достоверно ниже в сравнении с группами пациентов с БА, СРК и ЯК ( p ≤ 0.01 во всех случаях); при этом данный показатель в трех выборках больных стати- стически значимо не различался.

Результаты секвенирования и классификации полу- ченных ридов представлены в табл. 1. Все библиотеки содержали более 100 тыс. ридов, в среднем более 99.6 % ридов таксономически отнесены к определенному типу бактерий. В двух образцах больных БА и одном образце пациента с СРК обнаружены последовательности архей (тип Euryarchaeota).

Альфа-разнообразие бактериальных сообществ в исследуемых группах оценивали с помощью индекса Шен- нона – комплексного показателя, учитывающего количе- ство видов и их выровненность. Самым низким индекс Шеннона был для выборки ЯК, самым высоким – для ЗД (рис. 1), хотя различия не имели статистической значи- мости. Оценка выборок методом главных компонентов показала, что наиболее компактно расположены точки, характеризующие библиотеки последовательностей из группы ЗД (рис. 2). Расположение точек, характеризующих библиотеки из выборок пациентов, при меньшем значении индекса Шеннона по сравнению с таковым для ЗД свидетельствует об относительной нестабильности микробиомов у таких больных.

**Fig. 1.**
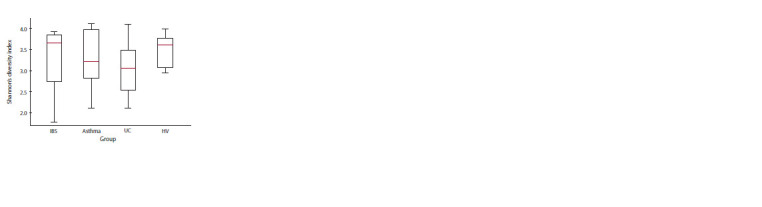
Shannon index, reflecting the alpha-diversity of the gut microbiome in samples obtained from patients with irritable bowel syndrome (IBS), bronchial asthma, ulcerative colitis (UC), and healthy volunteers (HV). Median values and quartiles are indicated.

**Fig. 2.**
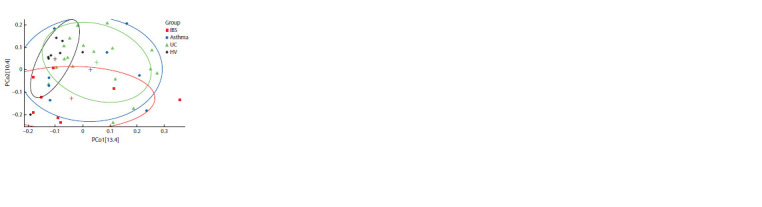
Visualization of sequence library analysis by the method of principal coordinates PCoA based on matrices of distances. Red squares indicate data for libraries from IBS samples; blue dots, from asthma samples; green triangles, from UC samples; and black diamonds – from HV samples. Plus signs of the corresponding colors mark the centers of areas occupied by the respective groups of samples. The values of the first and second principal coordinates are presented on the X and Y axes, respectively

Всего в библиотеках выявлены последовательности 12 типов бактерий (см. табл. 1); последовательности девяти типов присутствовали во всех четырех выборках. Из них последовательности пяти типов (Firmicutes, Bacteroidetes, Proteobacteria, Actinobacteria и Fusobacteria) определены во всех библиотеках, а последовательности, относящиеся к типу Verrucomicrobia, обнаружены во всех библиотеках из групп БА и ЗД, при этом в СРК и ЯК они отсутствовали в одной и четырех библиотеках соответственно. Последовательности представителей типов Tenericutes, Epsilonbacteraeota, Cyanobacteria, Synergistetes, Patescibacteria и Fibrobacteres найдены не во всех библиотеках, а последних трех типов – не во всех вы- борках. В среднем во всех группах доминировали после- довательности Firmicutes (в среднем по выборке > 53 %) и Bacteroidetes (в среднем > 18 %), причем в группе СРК последовательности Bacteroidetes выявлены статистиче- ски значимо чаще, чем в БА (в среднем 38.5 против 18.5 %; p ≤ 0.05). Третьими по встречаемости во всех выборках были последовательности Proteobacteria. Из табл. 1 видно, что меньше всего таких последовательностей зафиксировано в группе ЗД (1.36 %), в остальных выборках таких последовательностей отмечено существенно больше (СРК – 5.71 %, БА – 13.35 %, ЯК – 10.00 %); статистиче- ская достоверность различий показана даже для групп ЗД и СРК. Последовательности типа Actinobacteria были в среднем четвертыми по встречаемости, однако в выборках ЯК и СРК (заболевания кишечника) их встречаемость в среднем превышала таковую для последовательностей Proteobacteria в группе ЗД.

**Table 1.**
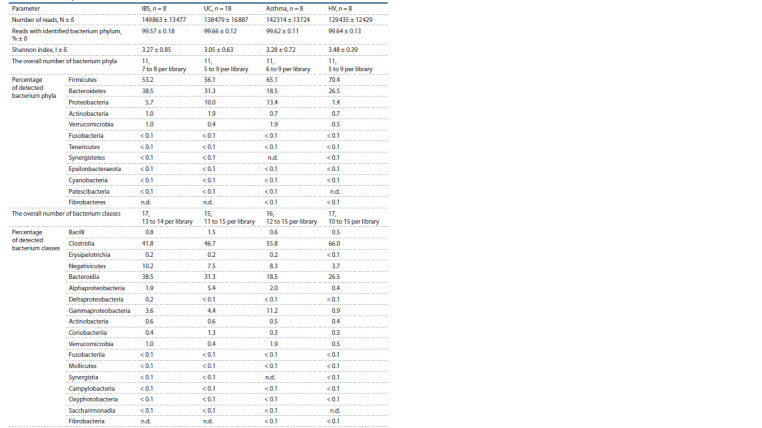
The taxonomy of reads in 16S rRNA libraries Notе. Library designations here and below: IBS, irritated bowel syndrome; UC, ulcerative colitis, HV, healthy volunteers. n.d., not detected.

Девять из двенадцати обнаруженных бактериальных типов представлены последовательностями единствен- ного класса, включая один доминирующий тип Bacteroidetes (класс Bacteroidia). Еще один доминирующий тип, Firmicutes, был представлен последовательностями четырех классов – Bacilli, Clostridia, Erysipelotrichia, Negativicutes, причем во всех выборках доминировали по- следовательности класса Clostridia. Тип Proteobacteria содержал последовательности трех классов – Alphaproteobacteria, Deltaproteobacteria и Gammaproteobacteria, у пациентов из этого типа преобладали последовательно-сти последнего класса. Тип Actinobacteria включал последовательности двух классов – Actinobacteria и Coriobacteriia. Всего выявлены последовательности 18 классов (см. табл. 1). Встречаемость последовательностей основ- ных порядков показана на рис. 3.

**Fig. 3.**
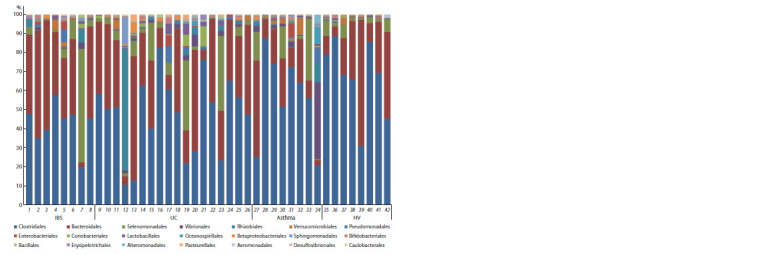
Taxonomic classification of operational taxonomic units at the order level based on the Silva v.132 (full) database. Samples 1–8 were obtained from patients with IBS; 9–26 from patients with UC; 27–34 from patients with bronchial asthma; and 35–42 from healthy volunteers (HV). Orders with abundance exceeding 0.1 % are shown.

Сравнительный анализ таксономического состава микробных сообществ

Известно, что основными компонентами микробного сообщества кишечника здоровых людей выступают представители типов Firmicutes (~70 %), Bacteroidetes (~30 %), Proteobacteria (< 5 %), Actinobacteria (< 2 %), Verrucomicrobia (< 1 %) и Fusobacteria (< 1 %); представители еще 10–12 типов бактерий являются транзиентными или встречаются в зависимости от региона проживания чело- века, его возраста и типа питания (Belizário et al., 2018). Анализ таксономического состава библиотек из выборки ЗД показал соответствие средних значений приведенным показателям (см. табл. 1). Это позволило оценить сдвиг таксономического состава в кишечном микробиоме пациентов с СРК и ЯК (заболевания кишечника) и БА от- носительно кишечного микробиома здоровых доноров.

Изменение соотношения количества представителей Firmicutes и Bacteroidetes в микробиоте кишечника мо- жет быть одним из индикаторов нарушения состояния микробиоты, при этом сдвиг этого соотношения как в сторону увеличения, так и уменьшения описан при ряде заболеваний (Tana et al., 2010; Rajilić-Stojanović et al., 2011; Jeffery et al., 2012; Jalanka-Tuovinen et al., 2014; Pozuelo et al., 2015; Tap et al., 2017). Соотношения Firmicutes/ Bacteroidetes в группе ЗД составило 2.6, в СРК и ЯК это соотношение существенно уменьшилось (1.4 и 1.8 соответственно), а в выборке БА – увеличилось (3.5) (табл. 2). Полученные данные согласуются с описанным ранее уменьшением встречаемости Firmicutes при ЯК (Machiels et al., 2014) и СРК (Jalanka-Tuovinen et al., 2014; Bennet et al., 2015; Pozuelo et al., 2015; Su et al., 2018), однако относительно СРК результаты разнятся и в ряде исследований зарегистрировано увеличение соотноше- ния Firmicutes/Bacteroidetes (Tana et al., 2010; Rajilić- Stojanović et al., 2011; Jeffery et al., 2012; Tap et al., 2017).

Обнаруженное в данном исследовании уменьшение доли последовательностей, принадлежащих типу Firmicutes, в выборках больных относительно группы ЗД наиболее выраженно среди пациентов с заболеваниями кишечника. Наибольший вклад в эти различия внесли последовательности класса Clostridia, уменьшение доли которых в выборках СРК и ЯК было статистически значи- мым ( p < 0.01 и p < 0.05 соответственно). Одновременно доля последовательностей класса Negativicutes во всех группах больных, наоборот, увеличилась (см. табл. 1). Ранее отмечено, что при СРК уменьшается доля после- довательностей семейств Ruminococcaceae и Erysipelotrichaceae, представители которых участвуют в продукции короткоцепочечных жирных кислот (Załęski et al., 2013; Pozuelo et al., 2015).

В данном исследовании также выявлено снижение доли последовательностей Ruminococcaceae как при СРК, так и при ЯК, однако доля последовательностей Erysipelotrichaceae в выборках пациентов с заболеваниями кишечника и БА существенно не изменилась по сравнению с ЗД (см. табл. 2). Среди последовательностей Ruminococcaceae особенно заметно снижение доли по- следовательностей Faecalibacterium spp. в группе СРК относительно ЗД ( p < 0.01), что соответствует получен- ным ранее результатам (Rajilić-Stojanović et al., 2011; Rodiño- Janeiro et al., 2018). Кроме этого, следует отме- тить заметное увеличение доли последовательностей сем. Veillonellaceae среди больных, однако описанное в литературе увеличение доли последовательностей Veillonella spp. при СРК (Malinen et al., 2005; Tana et al., 2010; Rigsbee et al., 2012) в нашей работе не определено, хотя при ЯК наблюдалось. Не обнаружено существенных отличий встречаемости во всех исследуемых выборках последовательностей Staphylococcus spp., Enterococcus spp. и Streptococcus spp., среди которых присутствуют патогенные и условно-патогенные виды. Зафиксировано существенное уменьшение доли последовательностей Roseburia spp. и Lachnospira spp. в группе БА (см. табл. 2). Ранее показано, что Roseburia spp. наряду с представителями родов Bif idobacterium и Lactobacillus явля- ется основным продуцентом полиненасыщенных жир-ных кислот, уменьшение концентрации которых в ки- шечнике детей раннего возраста ассоциировано с рис- ком развития БА (Chiu et al., 2019; Lee-Sarwar et al., 2020). Снижение числа представителей рода Lachnospira связано с увеличением относительного обилия представителей рода Clostridium, что, в свою очередь, также ассоциировано с повышением риска развития астмы у детей (Arrieta et al., 2015; Stiemsma et al., 2016; Hufnagl et al., 2020).

**Table 2.**
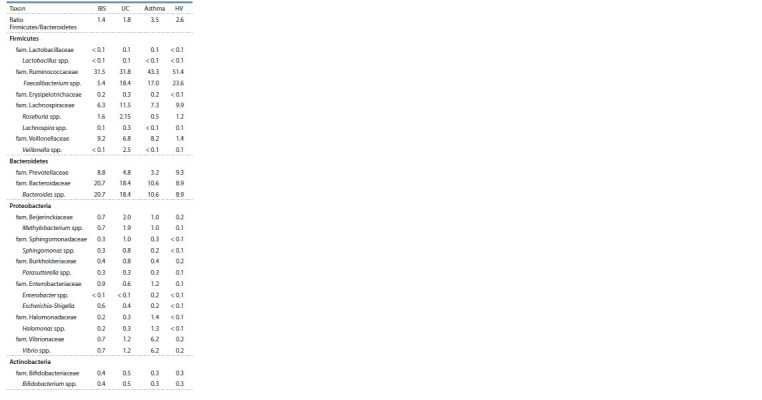
Mean percentages of sequences from different bacterial taxa in 16S rRNA libraries

В литературе описано уменьшение доли последовательностей, принадлежащих типу Bacteroidetes у паци- ентов с ЯК (Machiels et al., 2014). В нашем исследовании средняя доля последовательностей этого типа в группах ЯК и СРК, наоборот, несколько увеличилась по сравнению с ЗД (см. табл. 1), причем наиболее значимое увели- чение зарегистрировано для последовательностей Bac-teroides spp. (см. табл. 2). В выборке БА доля последо- вательностей Bacteroidetes незначительно уменьшилась по сравнению с ЗД, однако встречаемость последова- тельностей Bacteroides spp. в этой группе значимо не изменилась. Следует отметить, что в выборках СРК, ЯК и БА заметно увеличилось соотношение представителей семейств Bacteroidaceae/ Prevotellaceae по сравнению с ЗД (2.4, 3.8, 3.3 против 0.9 соответственно). Обычно доля последовательностей Bacteroidaceae больше у людей, питающихся преимущественно белковой пищей, а доля Prevotellaceae – у людей, отдающих предпочтение рас- тительной пище: жителей средиземноморского региона, Юго-Восточной Азии и у вегетарианцев (Ley, 2016). До- минирующие виды из обоих семейств, обнаруживаемые в кишечной микробиоте человека, способны расщеплять различные растительные полисахариды (Flint et al., 2012), и для объяснения различий в исследуемых выборках не- обходима видоспецифическая характеризация образцов

Значительные отличия обнаружены во встречаемости в исследуемых группах последовательностей типа Proteobacteria, ряд представителей которого относятся к патогенным и условно-патогенным бактериям. В выборках СРК, ЯК и БА по сравнению с ЗД вклад таких после- довательностей был существенно выше (см. табл. 1), что коррелировало с описанными ранее данными для пациентов с СРК и ЯК (Saebo et al., 2005; Gradel et al., 2009; Sonnenberg, Genta, 2012; Bennet et al., 2015; Shin et al., 2015; Shen et al., 2018). При этом в группах пациентов заметно увеличилась доля последовательностей классов Alphaproteobacteria и Gammaproteobacteria: в частности, обнаружен рост встречаемости последовательностей ро- дов Methylobacterium (сем. Beijerinckiaceae), Sphingomonas (сем. Sphingomonadaceae), Parasutterella (сем. Burkholderiaceae), Halomonas и Vibrio (сем. Halomonadaceae), а так-же последовательностей Escherichia spp. и Shigella spp. В выборке БА обнаружено значимо больше последова- тельностей рода Enterobacter (сем. Enterobacteriaceae). Важно отметить, что некоторые виды вышеперечислен- ных родов могут вызывать инфекции, особенно у людей со сниженным иммунитетом (Lai et al., 2011; Kovaleva et al., 2014; Baker-Austin et al., 2018; Chen et al., 2018). Более того, доказана ассоциация Parasutterella, в част- ности P. excrementihominis, с СРК и воспалительными заболеваниями кишечника (Shin et al., 2015; Chen et al., 2018). У пациентов по сравнению с ЗД не зафиксировано заметного увеличения последовательностей родов протеобактерий, членами которых являются хорошо известные инфекционные агенты: Klebsiella, Proteus, Salmonella, Serratia (все сем. Enterobacteriaceae), Acinetobacter (сем. Moraxellaceae), Pseudomonas (сем. Pseudomonadaceae) и Stenotrophomonas (сем. Xanthomonadaceae). Несмотря на отсутствие заметного повышения доли последовательностей вышеперечисленных родов в группах СРК, ЯК и БА относительно ЗД факт увеличения встречаемости после- довательностей Proteobacteria, в частности, Enterobacteriaceae, в исследуемых выборках библиотек от пациентов служит признаком дисбиоза кишечной микрофлоры (Shin et al., 2015).

Доля последовательностей типа Actinobacteria в груп- пах ЯК и СРК в среднем превышала таковую для ЗД (см. табл. 1), однако вклад последовательностей Bif idobacterium spp. во всех четырех исследуемых выборках практически не различался (см. табл. 2), что не соот- ветствует результатам, описанным ранее для пациентов с СРК (Malinen et al., 2005; Rajilić-Stojanović et al., 2011; Parkes et al., 2012; Zhuang et al., 2017). Bif idobacterium spp. наряду с Lactobacillus spp. (сем. Bacilli) способны модулировать состав кишечной микробиоты и влиять на иммунную систему человека путем взаимодействия с рецепторами CD209, экспонированными на поверхности дендритных клеток (Pace et al., 2015). Кроме того, отдель- ные представители этих родов секретируют бактериоцины – соединения, обладающие бактерицидным эффектом в отношении ряда патогенных бактерий (Angelakis et al., 2013; Rodiño-Janeiro et al., 2018). Отличий во встречае- мости последовательностей лактобацилл в исследуемых группах также не обнаружено

## Заключение

В данном пилотном исследовании проведена сравнитель- ная оценка биоразнообразия и таксономической струк- туры 16S-профилей кишечного микробиома пациентов с СРК, ЯК и БА и здоровых добровольцев. Подтверждены различия между кишечными сообществами бактерий у пациентов с воспалительными заболеваниями кишечника и таковыми у здоровых людей. В ряде случаев полученные нами данные согласуются с результатами аналогичных зарубежных исследований: уменьшение индекса Шен- нона, соотношения и обилия представителей Firmicutes/ Bacteroidetes и увеличение доли последовательностей Proteobacteria в микробиоме пациентов с воспалитель- ными заболеваниями кишечника в отличие от здоровых. Вместе с тем по некоторым показателям наши результаты отличаются от опубликованных ранее. Так, у пациентов с СРК и ЯК не уменьшилась доля последовательностей Erysipelotrichaceae, а даже несколько увеличилась (пред- ставители этого семейства продуцируют короткоцепочеч- ные жирные кислоты); не отмечено и уменьшение встреча- емости лакто- и бифидобактерий (отвечают за продукцию полиненасыщенных жирных кислот). Также нетипично увеличение доли последовательностей Bacteroidetes, в частности Bacteroides spp., у больных ЯК и СРК вместо зарегистрированного ранее уменьшения (Machiels et al., 2014; Dubinsky, Braun, 2015); не обнаружен рост доли последовательностей Veillonella spp. при СРК. Подоб- ные отличия могут быть объяснены как многократными попытками лечения пациентов, кишечные сообщества которых изучали, так и региональными характеристиками популяции и особенностями питания.

Следует отметить, что ранее мы описывали похожую выборку больных ЯК из Западной Сибири (Тикунов и др., 2020) и в целом данные по долям последователь- ностей из семейств, принадлежащих типам Firmicutes, Bacteroidetes и Actinobacteria, в обоих исследованиях существенно не различались; лишь доля последовательно- стей Proteobacteria в предыдущей работе была несколько меньше. Также в настоящем исследовании, в отличие от предыдущего, у пациентов с ЯК не обнаружены последо- вательности архей. Отсутствие существенных различий между 16S-профилями кишечных микробиомов двух групп больных ЯК свидетельствует об определенных ре- гиональных особенностях микробиоты таких пациентов. Вместе с тем в данное пилотное исследование включены лишь по 8, 18 и 8 образцов пациентов с СРК и ЯК и здоровых участников соответственно. Для вывода о том, являются ли обнаруженные тенденции закономерностями, необходимо продолжение исследований с использованием большего количества анализируемых образцов.

При этом ряд результатов исследования бактериальных сообществ пациентов с БА получены впервые. Выпол- ненные до настоящего времени исследования включали изучение микробиоты лишь младенцев и детей постарше с БА, причем большая часть работ посвящена микробиоте дыхательных путей и ротовой полости. Исследования же содержимого кишечника детей с БА в основном сводились к поиску ассоциаций между содержанием определенных соединений и развитием астмы. В нашей работе оценены бактериальные сообщества кишечника взрослых паци- ентов с БА. В связи с этим нельзя сделать заключение о том, насколько существенно обнаруженное увеличение соотношения Firmicutes/Bacteroidetes в выборке БА от- носительно ЗД. Ряд выявленных особенностей кишечной микробиоты взрослых с БА согласуются с описанными ранее показателями таковой у больных детей. Так, в кишечной микробиоте взрослых с БА, как и детей, опре- делено уменьшение доли последовательностей Roseburia spp., Lachnospira spp., Veillonella spp., однако доля после- довательностей Faecalibacterium spp. и Lactobacillus spp. у взрослых пациентов, в отличие от детей, была такой же, как и у здоровых. Возможно, недостаточную пред- ставленность некоторых, но не всех бактерий в кишечной микробиоте детей с БА с годами компенсирует диета или прием пробиотиков. Также наблюдаемые различия могут быть связаны не с возрастом пациентов, а региональными особенностями питания. Выявленное в данном исследо- вании существенное превышение доли последователь- ностей Enterobacteriaceae в выборке БА по сравнению с ЗД закономерно, так как свидетельствует о дисбиозе кишечной микробиоты. Однако отсутствует достоверное объяснение обнаруженного нами значимого превышения доли последовательностей Halomonas и Vibrio в кишечной микробиоте пациентов с БА по сравнению не только со здоровыми добровольцами, но и больными СРК и ЯК. Важно, что указанные различия не связаны с особен- ностями питания пациентов с БА, поскольку доля после- довательностей Halomonas spp. и Vibrio spp. у больных атопическим дерматитом, находившихся одновременно с пациентами с БА в том же отделении медицинского уч- реждения, не отличалась от таковой в группах ЗД, СРК и ЯК. Вышеперечисленные факты свидетельствуют о том, что дисбаланс кишечной микрофлоры, наблюдаемый при БА, ассоциирован с развитием этого заболевания, однако для понимания роли отдельных компонентов микробио- ты кишечника в его патогенезе необходимы дальнейшие исследования с включением более объемных выборок.

## Conflict of interest

The authors declare no conflict of interest.
